# Early diagnosis of AKI in the ICU: urinary chitinase 3-like protein 1 as a novel renal troponin

**DOI:** 10.1186/2197-425X-3-S1-A840

**Published:** 2015-10-01

**Authors:** J De Loor, L De Crop, C Clauwaert, S Bracke, D Vermeiren, K Demeyere, E Meyer, E Hoste

**Affiliations:** Ghent University, Laboratory of Biochemistry, Department of Pharmacology, Toxicology and Biochemistry, Faculty of Veterinary Medicine, Merelbeke, Belgium; Ghent University, ICU, Ghent University Hospital, Faculty of Medicine and Health Sciences, Ghent, Belgium

## Introduction

Our group recently validated urinary chitinase 3-like protein 1 (**UCHI3L1**) as novel biomarker for acute kidney injury (**AKI**) in septic mice [1].

## Objectives

This ensuing study aimed to investigate whether our preclinical finding could be translated to humans and whether UCHI3L1 performed equally to the AKI biomarker urinary neutrophil gelatinase-associated lipocalin (**UNGAL**) [2].

## Methods

Prospective cohort study at the surgical and medical ICUs of the University Hospital Ghent from Sept. 2012 till Aug. 2014. Patients were **included if**: age ≥18 y; arterial and urinary catheter present; expected ICU stay ≥48 h; and respiratory or cardiovascular SOFA score ≥2 resp. ≥1. Participation was **excluded if**: AKI KDIGO_Full_ stage ≥2 at inclusion; chronic kidney disease stage 5; or no written informed consent.

Blood and urine were collected at inclusion. Each patient was sampled a 2^nd^ time at 6 pm if the 1^st^ collection was before noon, then at 6 am and pm on days 2-4, and at 6 am on days 5-7. The study stopped if the patient was discharged from the ICU before day 7. Reference serum creatinine (SCr) was defined as the lowest SCr value within the last 3 months prior to enrollment.

The **primary endpoint** was **AKI KDIGO**_**Full**_**stage ≥2** within **12 h** after enrollment. Secondary endpoints were: AKI KDIGO_Full_ stage ≥2 within 24 h and 7 d after enrollment; and AKI KDIGO_SCr_ stage ≥2 within 12 h, 24 h and 7 d after enrollment.

## Results

In total 181 patients were included, of which 6 (**3%**) reached the primary endpoint. Baseline characteristics showed no differences with the exception of age (70.5 y [IQR: 65.8-78.0] vs. 59.0 [50.0-70.0] for endpoint pos. resp. neg.; P = 0.040). At ICU admission, the only significant difference was the proportion of patients referred from another department (66.7 vs. 22.3% for endpoint pos. resp. neg.; P = 0.029).

**Figure 1:**
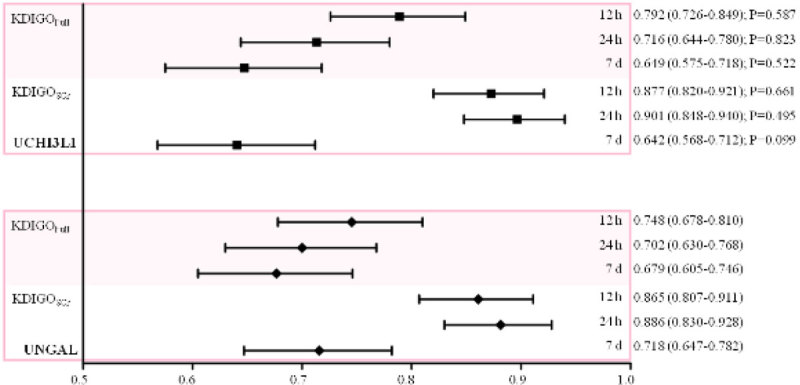
Figure 1

Both UCHI3L1 and UNGAL measured at inclusion were good predictors of the primary endpoint, with an AUC-ROC of 0.792 (95% CI: 0.726-0.849) resp. 0.748 (0.678-0.810). The difference between both areas was not significant (P = 0.587). Results for all endpoints are shown in Figure 1.

## Conclusions

UCHI3L1 was a valuable diagnostic biomarker for moderate or severe AKI in this adult ICU cohort, and performed similar to UNGAL.

## Grant Acknowledgment

FWO grant to De Loor J. IOF grant to Meyer E. and Hoste E.

Patent: US2014006991 and EP201211163. Valorisation: bimetra@uzgent.be
